# Pulmonary and functional hallmarks after SARS-CoV-2 infection across three WHO severity level-groups: an observational study

**DOI:** 10.3389/fmed.2025.1561387

**Published:** 2025-04-07

**Authors:** Patrícia Blau Margosian Conti, Maria Ângela Gonçalves Oliveira Ribeiro, Carla Cristina Souza Gomez, Aline Priscila Souza, Daniela Souza Paiva Borgli, Eulália Sakano, Mauro Alexandre Pascoa, Silvana Dalge Severino, Tayná Castilho, Fernando Augusto Lima Marson, José Dirceu Ribeiro, UNICOVID Study Group, Adyléia Aparecida Dalbo Contrera Toro, Aline Cristina Gonçalves, Andrea de Melo Alexandre Fraga, André Moreno Morcillo, Andressa Oliveira Peixoto, Andrei Carvalho Sposito, Bianca Aparecida Siqueira, Emília Silva Gonçalves, Emília Raposo Nascimento, Gil Guerra Junior, Jenniffer Tayna Orzechowski Furtado, Lucas Rodrigues de Moraes, Maria de Fatima Corrêa Pimenta Servidoni, Mariana Zorron, Marcos Tadeu Nolasco da Silva, Maíra Seabra Assumpção, Milena Baptistela Grotta, Renan Marrichi Mauch, Simone Appenzeller, Thiago Luís Infanger Serrano

**Affiliations:** 1 Department of Pediatrics, School of Medical Sciences, University of Campinas, Campinas, Brazil; 2 Department of Ophthalmology-Otorhinolaryngology, School of Medical Sciences, University of Campinas, Campinas, Brazil; 3 LunGuardian Research Group, Epidemiology of Respiratory and Infectious Diseases, Postgraduate Program in Health Sciences, São Francisco University, Bragança Paulista, Brazil; 4 Laboratory of Molecular Biology and Genetics, Postgraduate Program in Health Sciences, São Francisco University, Bragança Paulista, Brazil; 5 Laboratory of Clinical Microbiology and Genetics, Postgraduate Program in Health Sciences, São Francisco University, Bragança Paulista, Brazil

**Keywords:** 6-min walk test, chest computed tomography, fractional exhaled nitric oxide, hand grip strength, impulse oscillometry, lung function, maximum expiratory pressure, maximum inspiratory pressure

## Abstract

**Background:**

The manifestations of severe acute respiratory syndrome coronavirus 2 (SARS-CoV-2) infection range from flu-like symptoms to severe lung disease. The consequences of this inflammatory process impact overall function, which can be detected through both short- to long-term assessments. This study aimed to assess the pulmonary functional and structural characteristics of post-SARS-CoV-2 infection in patients with mild/moderate, severe, and critical clinical presentations.

**Methods:**

An observational, analytical, and cross-sectional study was conducted between 2020 and 2022, including participants with a confirmed diagnosis of coronavirus disease (COVID)-19, with mild/moderate (G1), severe (G2), and critical (G3) clinical presentations, all evaluated at least 3 months after acute infection. Spirometry, impulse oscillometry, fractional exhaled nitric oxide (FeNO), chest computed tomography, the 6-min walk test (6MWT), hand grip strength, maximum inspiratory pressure, and maximum expiratory pressure were assessed.

**Results:**

We enrolled 210 participants aged 18–70 years, 32.6% of whom were male, with older age observed in G3. The participants were grouped as follows: G1 (42.3%), G2 (25.7%), and G3 (31.9%). Percentage of predicted X5 differed between G1 and G2, being higher in G1. The percentage of predicted forced vital capacity (FVC) according to the Global Lung Function Initiative and its z-score were higher in G1. The FVC by Pereira was lower in G3 compared to G1. The percentage of predicted forced expiratory volume in 1 s (FEV_1_) by Pereira was also lower in G3. The Tiffeneau (FEV_1_/FVC) index was different among groups, increasing with disease severity. The percentage of predicted forced expiratory flow rate at 25–75% (FEF_25-75%_) of the FVC and FeNO were both higher in G2 than G1. Chest computed tomography revealed the presence of interstitial abnormalities, associated with disease severity. The respiratory muscle strength evaluation showed an association between higher maximum expiratory pressure values in G3 compared to G1, but no association with maximum inspiratory pressure was observed. The 6MWT distance covered decreased with increasing severity, with a lower percentage of predicted values in G3 compared to G1. The right-hand grip strength was also lower in G3 compared to G1.

**Conclusion:**

Alterations in pulmonary and functional markers were observed in post-COVID-19 evaluations, increasing with disease severity, as seen in G2 and G3. These findings highlight the complexity of post-COVID-19 functional assessments, given the long-term pulmonary sequelae and the consequent impairment of functional capacity.

## Graphical abstract

A comprehensive summary of the study’s key findings. Disease severity was determined by a physician, according to World Health Organization criteria. The G1 phenotype was characterized by mild to moderate clinical symptoms, with possible radiological findings or the presence of pneumonia on chest computed tomography. The G2 phenotype was characterized by respiratory distress and/or hypoxia/hypoxemia, while the G3 phenotype involved respiratory failure requiring mechanical ventilation, shock, and/or multiorgan dysfunction (5,16). 6-min walk test (6MWT); Coronavirus disease (COVID)-19 (COVID-19); Computed tomography (CT); Forced vital capacity (FVC); Forced expiratory volume in the first second (FEV_1_); Fractional exhaled nitric oxide (FeNO); Percentage of forced expiratory flow from 25 to 75% of FVC (FEF_25-75%_). 
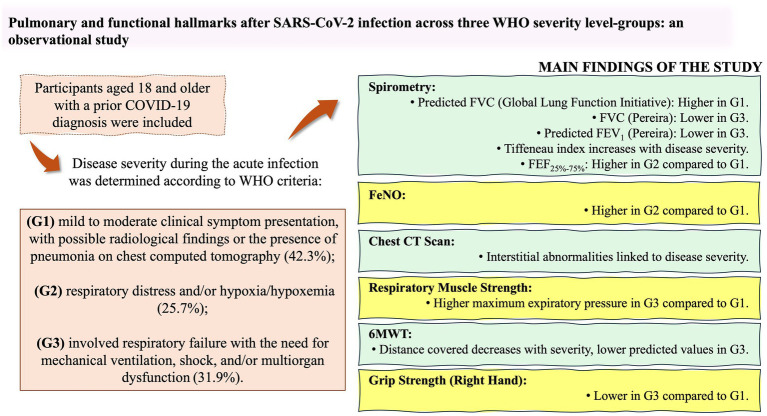

## 1 Introduction

The severe acute respiratory syndrome coronavirus 2 (SARS-CoV-2) infection led to the declaration of coronavirus disease (COVID)-19 as a pandemic by the World Health Organization (WHO), due to its high infectivity, transmission rate, and community mortality (1–4). Clinical manifestations range from flu-like symptoms to severe lung disease, and severity is classified by the WHO based on clinical symptoms and radiological findings. Older age, higher viral load, impaired immunity, preexisting comorbidities, and worse hospital admission scores have been described in previous studies as risk factors for disease severity, associated with worse outcomes, including intensive care unit (ICU) admission, prolonged hospitalization, functional sequelae, and increased morbidity and mortality (2, 5).

The virus infects the host through the respiratory tract, invading the epithelial cells and then entering the bloodstream, triggering an acute inflammatory response with a cytokine storm, which can lead to acute respiratory distress syndrome, with a potential need for mechanical ventilation in severe cases, contributing to lung injury, reduced diffusion capacity, and further impairment (6, 7). Additionally, other organs that express angiotensin-converting enzyme 2 receptors may become involved, leading to extrapulmonary manifestations that add an extra layer of complications involving cardiovascular, digestive, renal, nervous, endocrine, hematologic, and other systems (2, 6). The consequences of this inflammatory process are reflected in medium-term and long-term functional outcomes, with impacts on quality of life and society, affecting the public health system (6, 8–10). Long COVID-19 studies vary widely, due to differences in sampling, participant demographics, inclusion and exclusion criteria, methodologies, and even the definition itself (11–14). Additionally, the persistence of symptoms may differ among severity groups (12). Overall, the most widely accepted concept at present is that long COVID-19 should be defined as symptoms of the disease that persist for 3 months or more after the diagnosis of COVID-19 with an acute clinical phenotype of the disease (12).

Non-invasive evaluation of the respiratory system, peripheral muscle strength, and exercise capability and tolerance can be applied in outpatient settings and provide valuable information for disease management, treatment, rehabilitation, and public health practices. Therefore, this study aimed to assess pulmonary functional and structural characteristics, along with functional capacity, in patients with mild/moderate (G1), severe (G2), and critical (G3) clinical presentations, at least 3 months after their recovery from SARS-CoV-2 infection.

## 2 Methods

### 2.1 Study design

An observational, analytical, and cross-sectional study was conducted between 2020 and 2022 in the Laboratory of Pulmonary Physiology at the Center of Pediatrics Research at the University of Campinas. The study adhered to the Helsinki Declaration and the Ministry of Health guidelines and was approved by the University’s Ethics Committee, protocol no. 4.333.741. Written informed consent was obtained from all participants. We also followed the University’s Crisis Committee guidelines, which required all research staff involved in the study to have received a minimum of two doses of the COVID-19 vaccine.

Our primary aim was to stratify the long-term effects of COVID-19 on lung function and structure across three groups, namely, G1, G2, and G3. Our secondary aim was to gather and apply a set of standard assessment tools to perform a comprehensive follow-up assessment, capable of detecting abnormalities and referring participants to specialized care. This study complies with the Strengthening the Reporting of Observational Studies in Epidemiology (STROBE) guidelines (15).

#### 2.1.1 Participants of the study

This study was advertised through posters displayed in primary healthcare units in Campinas, at the University of Campinas’ Clinical Hospital, and on social media. We included participants aged 18 years and older who expressed interest in participating, had a confirmed COVID-19 diagnosis (based on clinical symptoms and laboratory tests as described below), signed informed consent, and had no prior diagnosis of chronic diseases. Participants with a history of lung or heart diseases (such as cystic fibrosis, pulmonary fibrosis, bronchiolitis obliterans, ciliary dyskinesia, bronchopulmonary dysplasia, congenital heart diseases, and congestive heart failure), exacerbated comorbidities (such as respiratory and cardiac diseases, diabetes mellitus, and arterial hypertension), or acute infectious disease (including COVID-19) were excluded from the study.

COVID-19 diagnosis was confirmed through molecular biology analysis using real-time polymerase chain reaction (PCR) to detect SARS-CoV-2 as well as immunological assessment with nasal and oropharyngeal swabs and/or serological examination of immunoglobulins (IgG, IgA, and IgM). Participants were included and evaluated at least 3 months after their acute COVID-19 diagnosis.

Disease severity was determined by a physician according to WHO criteria. The G1 phenotype was characterized by a mild-to-moderate clinical symptom presentation, with possible radiological findings or the presence of pneumonia on chest computed tomography (CT). The G2 phenotype was characterized by respiratory distress and/or hypoxia/hypoxemia, while the G3 phenotype involved respiratory failure with the need for mechanical ventilation, shock, and/or multiorgan dysfunction (5, 16).

All evaluations described below were performed simultaneously, except for the chest CT, which was scheduled for the following week. The vital signs measured for the inclusion and evaluation of the volunteers were systolic blood pressure, diastolic blood pressure, transcutaneous hemoglobin oxygen saturation, heart rate, respiratory rate, and temperature.

#### 2.1.2 Lung function tests

Spirometry and impulse oscillometry (IOS) were performed using Master Screen IOS (Erich Jaeger®, Germany), following the guidelines of the European Respiratory Society and the American Thoracic Society (ATS). Testing was conducted before and 15–20 min after the inhalation of 400 μg of Salbutamol. All data were presented as absolute values and percentages of predicted values, according to Pereira et al. and the Global Lung Function Initiative (GLI) (17–22).

The spirometry variables included in the study were as follows: (a) forced vital capacity (FVC), (b) forced expiratory volume in 1 s (FEV_1_), (c) Tiffeneau index (FEV_1_/FVC), (d) forced expiratory flow at 25% of FVC (FEF_25%_), (e) forced expiratory flow at 50% of FVC (FEF_50%_), (f) forced expiratory flow at 75% of FVC (FEF_75%_), and (g) forced expiratory flow between 25 and 75% of FVC (FEF_25–75%_). The IOS variables included in the study were as follows: (a) respiratory impedance (Z), (b) respiratory resistance (R), (c) reactance (X), (d) resonance frequency (Fres), and (e) reactance area (AX).

#### 2.1.3 Fractional exhaled nitric oxide test

Fractional exhaled nitric oxide (FeNO) was measured using the Analyzer CLD 88 series with DENOX 88 (Eco Medics™, Dürnten, Switzerland) according to the ATS recommendations. The mean value of two valid measurements in parts per billion (ppb) was used for statistical analysis. According to the literature, FeNO values greater than 25 ppb indicate a high possibility of eosinophilic airway inflammation (23).

#### 2.1.4 Chest computed tomography

Participants underwent a high-resolution chest CT using the GE Revolution EVO and Multislice Aquilion (Canon Medical Systems USA, Inc.), with 1.25 mm slices and tube currents ranging from 80 to 150 mA and at 100 kV. The assessment was performed during both inspiration and expiration. Images were displayed in a 1,500 HU (Hounsfield units) window and at −700 HU levels.

The images were assessed by two independent pneumologists, who were blinded to the participants’ clinical and laboratory data. Any discrepancies were resolved by a third party, a radiologist specialist, who determined the final score. Guidelines from the Brazilian Chest CT Illustrated Consensus (24) and the Fleischner Society (25) were applied to detect lung abnormalities, including ground-glass opacity, consolidation, linear atelectasis, bronchiectasis, and interstitial abnormalities (reticulation and thickening of the interalveolar septa).

Fibrotic interstitial abnormalities were characterized by structural distortion, traction bronchiectasis, and/or honeycombing. Non-fibrotic pulmonary interstitial abnormalities were identified by interstitial injury and ground-glass opacity. This method was adapted to describe idiopathic pulmonary fibrosis (26, 27). Air trapping was also assessed during expiration (28–30).

#### 2.1.5 Dynamometry

Hand grip strength was assessed using a digital dynamometer (Saehan^®^, Masanhoewon-gu, Changwon-si, Gyeongsangnam-do, Republic of Korea) (31, 32). The participant was seated with elbows flexed at 90°, holding the dynamometer. Six attempts were performed, alternating hands, following the guidelines of the American Society of Hand Therapists, the Brazilian Society of Upper Limb Therapy, and the International Federation of Societies for Surgery of the Hand (33, 34). The highest values obtained from both the right and left hands were selected for statistical analysis. The obtained values are expressed in absolute numbers and as a percentage of predicted values using the Brazilian equation published by Schlüssel et al. (33).

#### 2.1.6 6-min walk test

Two tests were performed following the ATS protocol, with a 15-min interval between them, until the patient recovered, and the best result was selected for statistical analysis. Heart rate, respiratory rate, peripheral oxygen saturation, and arterial blood pressure were measured, and the Borg scale for perceived exertion was applied. We analyzed the distance covered and the percentage of the predicted distance according to the standard and validated reference equations (35–37).

#### 2.1.7 Maximum inspiratory and expiratory pressures

Maximum inspiratory pressure (MIP) and maximum expiratory pressure (MEP) were measured during forced inspiration and expiration. Participants were instructed to breathe in tidal volume through the mouthpiece and then perform a briefly sustained maximal forced inspiration for the MIP measurement. For the MEP, participants inhaled to total lung capacity and then performed a maximal forced expiration, sustained for 1–3 s. The mouthpiece was connected to a vacuum manometer (Ger-Ar), with a scale ranging from −300 to +300 cmH_2_O (18). Three measurements were recorded, and the best value was selected. Respiratory muscle weakness was considered present when values were lower than 70% of the predictive value (35).

#### 2.1.8 Patient and public involvement

Patients and members of the public were not involved in the conceptualization, design, or data interpretation of this study. Recruitment was conducted through posters displayed in healthcare facilities, where individuals interested in participating contacted the research team. Results were provided to participants immediately after the evaluation, and those with abnormal findings were promptly referred for appropriate medical assistance.

### 2.2 Statistical analysis

Data were recorded using the university’s REDCap platform, and descriptive and inferential statistical analyses were performed using Statistical Package for Social Sciences software (IBM Corp. Released 2023. IBM SPSS Statistics for Macintosh, Version 29.0.2.0 Armonk, NY: IBM Corp). The results are presented as the mean and 95% confidence interval (or standard deviation) and/or median and 95% confidence interval (or interquartile range), depending on the normality (or lack thereof) of the data distribution obtained in the study. In addition, categorical data are presented as absolute (n) and relative (%) frequencies.

In our study, missing data imputation was performed for markers with less than 40% missing data. Imputation was carried out using XLSTAT Statistical Software^1^ for Excel (.xls). Specifically, quantitative data imputation was performed using the Markov Chain Monte Carlo algorithm, while qualitative data imputation was performed using the non-linear iterative partial least squares algorithm. The resulting dataset was then uploaded into SPSS software for descriptive and inferential statistical analyses. The missing data imputation process followed strict criteria based on the literature (36–40). No imputation was performed to infer the severity of patients as defined by the WHO criteria for COVID-19 severity.

**Figure 1:**
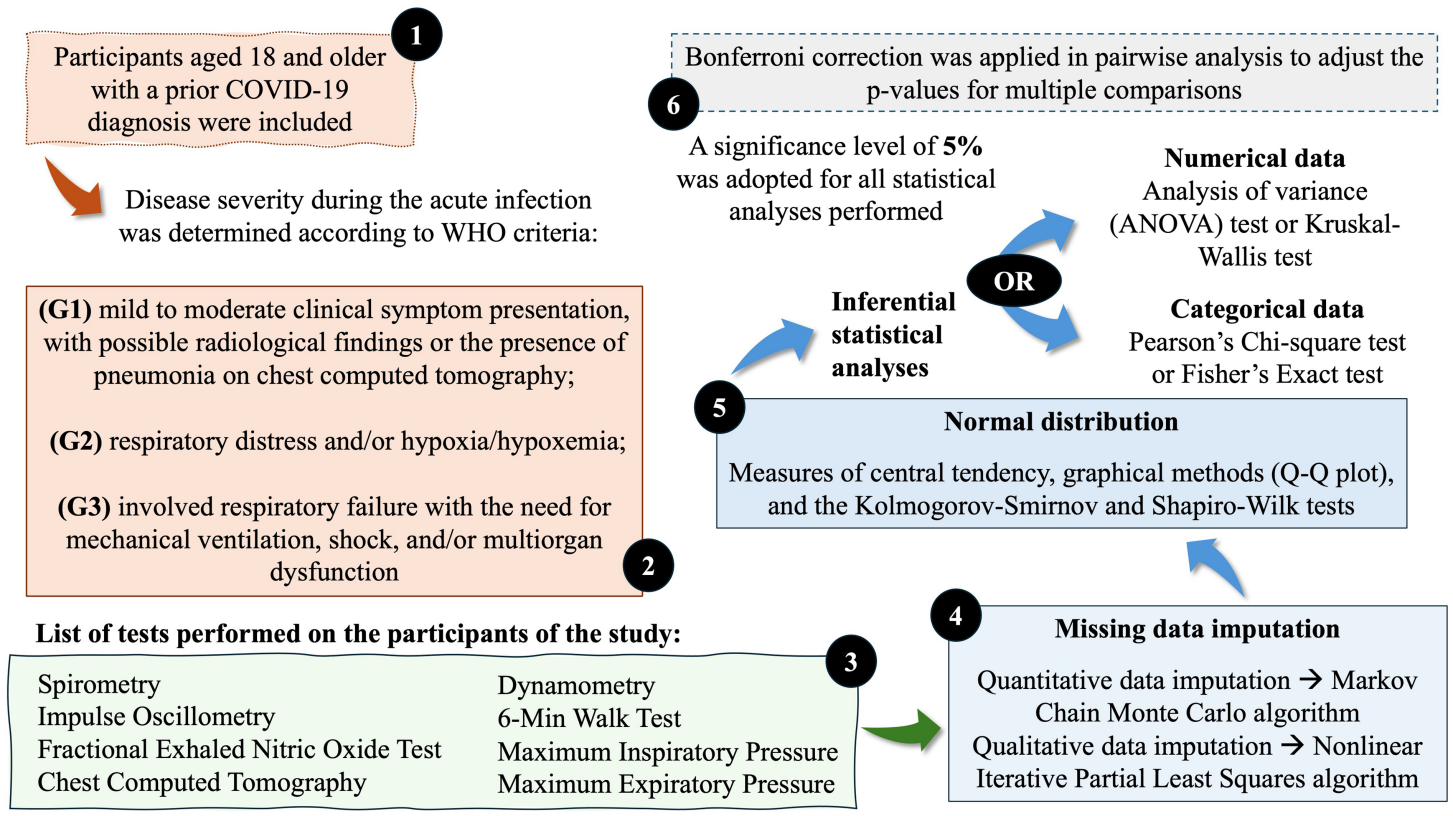
Complete representation of the study protocol. Disease severity was determined by a physician, according to the World Health Organization (WHO) criteria. The G1 phenotype was characterized by mild-to-moderate clinical symptoms, with possible radiological findings or the presence of pneumonia on chest computed tomography. The G2 phenotype was characterized by respiratory distress and/or hypoxia/hypoxemia, whereas the G3 phenotype involved respiratory failure requiring mechanical ventilation, shock, and/or multiorgan dysfunction (5, 16). Coronavirus disease (COVID)-19 (COVID-19).

Normal distribution was assessed using measures of central tendency, graphical methods (q-q plot), and the Kolmogorov–Smirnov and Shapiro–Wilk tests (Supplementary material). After analyzing the normality of the data, inferential statistical analyses were performed. In this context, analysis of variance (ANOVA) and the Kruskal–Wallis test were applied to assess the association between quantitative data and the severity of COVID-19, according to the WHO guidelines for parametric (normal distribution) and non-parametric (non-normal distribution) analysis, respectively (41–43). Bonferroni correction was applied in pairwise analysis to adjust the *p*-values for multiple comparisons between the groups of COVID-19 patients according to severity as evaluated using the WHO criteria. Categorical data were evaluated using Pearson’s chi-square or Fisher’s exact test; these tests were used to assess the association between chest CT scores, sex, hospitalization localization, and COVID-19 severity. For statistical purposes, mild and moderate phenotypes were grouped into G1. A significance level of 5% was adopted for all statistical analyses performed. A complete study protocol is presented in Figure 1.

## 3 Results

### 3.1 Demographical and clinical data

We enrolled 210 participants aged 18 to 70 years, of whom 100 (47.6%) were male, with a mean age of 48.09 ± 11.97 years. All vital signs measured at the time of the evaluation were within normal limits. A total of 89 (42.3%), 54 (25.7%), and 67 (31.9%) of participants were classified into G1, G2, and G3, respectively.

The percentage of male patients increased with the severity according to the WHO criteria, being reported in 29 (32.6%), 26 (50.0%), and 44 (65.7%) of the patients classified in the G1, G2, and G3 groups, respectively (*p*-value <0.001). In G2, 43 (79.6%) of the participants were hospitalized in general wards (*p*-value <0.001), and in G3, 59 (88.1%) of the participants were hospitalized in intensive care units (*p*-value <0.001). Age also differed among the severity groups, being higher in G2 and G3 (*p*-value <0.001). Demographics are described in Table 1.

**Table 1:** Distribution of demographic and clinical characteristics of the study participants according to coronavirus disease (COVID)-19 severity.

Markers	Groups	Clinical phenotype during the acute infection phase			Total	*P*-value
		Mild–moderate (G1) *N* = 89	Severe (G2) *N* = 54	Critical (G3) *N* = 67		
Sex*						
	Male	29 (32.6%)	27 (50.0%)	44 (65.7%)	100 (47.6%)	**<0.001**
	Female	60 (67.4%)	27 (50.0%)	23 (34.2%)	110 (52.4%)	
Age (years) **						
		44.11 ± 12.12	50.35 ± 12.03	51.58 ± 10.20	48.09 ± 11.97	**<0.001**
		43.0 [18.0]	52.0 [14.75]	52.0 [16.0]	49.0 [17.0]	
Hospitalization—general ward stay *,^a^						
	No	83 (93.3%)	11 (20.4%)	60 (89.6%)	154 (73.3%)	**<0.001**
	Yes	6 (6.7%)	43 (79.6%)	7 (10.4%)	56 (26.7%)	
Hospitalization—intensive care unit stay***,^b^						
	No	88 (98.9%)	48 (88.9%)	8 (11.9%)	144 (68.6%)	**<0.001**
	Yes	1 (1.1%)	6 (11.1%)	59 (88.1%)	66 (31.4%)	
Emergency room/primary healthcare unit stay ***,^c^						
	No	85 (95.9%)	52 (96.3%)	66 (98.5%)	203 (96.7%)	0.624
	Yes	4 (4.5%)	2 (3.7%)	1 (1.5%)	7 (3.3%)	

a The period during which patients are admitted to a regular hospital ward for monitoring and treatment of their clinical condition.

b The period during which patients are admitted to the intensive care unit for more intensive monitoring and treatment due to the severity of their clinical condition.

c The period during which patients are under care in the emergency room or primary healthcare unit for acute management or initial treatment before being discharged or transferred for further care.

*Pearson chi-square test; **Kruskal–Wallis test; ***Fisher’s exact test. The significant p-values are presented in bold type. The numerical data are displayed as mean ± standard deviation and median [interquartile range]. The categorical data are displayed as number of participants (N) and percentage (%). Disease severity was determined by a physician, according to the World Health Organization criteria. The G1 phenotype was characterized by mild-to-moderate clinical symptoms, with possible radiological findings or the presence of pneumonia on chest computed tomography. The G2 phenotype was characterized by respiratory distress and/or hypoxia/hypoxemia, whereas the G3 phenotype involved respiratory failure requiring mechanical ventilation, shock, and/or multiorgan dysfunction (5, 16).

### 3.2 Lung function tests

Table 2 describes the pulmonary function parameters for the severity groups assessed. The IOS evaluation showed that the percentage of predicted X5 significantly differed between G1 [103.17 (95%CI = 94.34–110.62)] and G2 [80.72 (95%CI = 71.14–96.67)], being higher in G1 (*p* = 0.007).

**Table 2:** Association between functional hallmarks assessed across different coronavirus disease (COVID)-19 severity groups.

Markers	Clinical phenotype during the acute infection phase			
	Mild–moderate (G1) *N* = 89	Severe (G2) *N* = 54	Critical (G3) *N* = 67	*P*-value
Impulse oscillometry system				
Z5	0.41 (0.38–0.45)	0.42 (0.39–0.49)	0.45 (0.40–0.48)	0.512 *
R5	0.39 (0.36–0.42)	0.40 (0.37–0.47)	0.42 (0.39–0.46)	0.472 *
R5%	125.03 (115.85–135.71)	106.96 (100.32–112.68)	115.78 (105.56–123.61)	0.073 *
R20	0.32 (0.30–0.367)	0.34 (0.31–0.36)	0.34 (0.32–0.37)	0.913 *
R20%	106.60 (102.61–118.46)	100.21 (95.92–106.86)	104.52 (97.10–115.27)	0.564 *
X5	0.12 (−0.14–0.11)	0.12 (−0.14–0.09)	0.12 (−0.14–0.11)	0.606 *
X5%	103.17 (94.34–110.62)	80.72 (71.14–96.67)	92.79 (90.88–103.29)	**0.010** ^**a**^,*
Fres	14.86 (13.48–16.27)	15.36 (14.66–17.87)	16.49 (14.92–17.45)	0.196 *
Fres%	118.74 (108.15–127.67)	118.80 (110.99–129.99)	122.67 (106.20–135.83)	0.917 *
AX	0.46 (0.39–0.62)	0.58 (0.41–0.75)	0.66 (0.44–0.79)	0.396 *
AX%	140.00 (115.31–181.25)	121.75 (101.32–175.90)	146.00 (106.67–211.11)	0.961 *
Spirometry				
FVC	3.70 (3.49–3.95)	3.80 (3.51–4.06)	3.80 (3.37–4.41)	0.818 *
FVC% GLI	105.03 (100.65–108.54)	103.87 (98.01–107.15)	97.53 (95.57–101.61)	**0.002** ^**b**^,*
zFVC GLI	0.34 (0.04–0.56)	0.15 (−0.17–0.35)	-0.15 (−0.29–-0.10)	**0.003** ^**c**^,*
FVC% Pereira	99.50 (95.50–103.30)	97.35 (93.00–101.10)	90.20 (88.50–93.70)	**<0.001** ^**d**^,*
FEV_1_	3.04 (2.86–3.18)	3.07 (2.77–3.32)	3.05 (2.74–3.36)	0.935 *
FEV_1_% GLI	103.50 (100.44–106.43)	102.57 (98.75–106.11)	98.13 (94.60–101.41)	0.065 **
zFEV_1_ GLI	0.25 (0.03–0.45)	0.21 (−0.05–0.46)	−0.13 (−0.36–0.08)	**0.046** ^**e**^,**
FEV_1_% Pereira	98.20 (94.00–101.10)	96.00 (93.69–102.30)	90.60 (86.80–97.05)	**0.008** ^**f**^,*
FEV_1_/FVC	0.81 (0.80–0.82)	0.82 (0.81–0.85)	0.82 (0.81–0.83)	0.159 *
FEV_1_/FVC% GLI	98.85 (96.93–100.64)	101.42 (100.21–103.05)	101.64 (100.30–103.77)	**<0.001** ^**g**^,*
zFEV_1_/FVC GLI	−0.17 (−0.40–0.08)	0.17 (−0.05–0.41)	0.21 (0.04–0.48)	**0.001** ^**h**^,*
FEV_1_/FVC% Pereira	80.70 (79.67–81.63)	83.90 (81.40–85.66)	81.51 (80.80–83.14)	**0.010** ^**i**^,*
FEF_25–75_	3.04 (2.69–3.23)	3.11 (2.91–3.55)	3.22 (3.05–3.68)	0.235 *
FEF_25–75_%pred	95.40 (87.70–101.70)	105.90 (101.70–116.01)	104.70 (98.00–111.50)	**0.021** ^**j**^,*
Fractional exhaled nitric oxide (FeNO) test				
FeNO test	16.00 (13.33–18.40)	20.35 (16.58–22.48)	19.66 (16.60–22.79)	**0.013** ^**k**^,*

The following significant p-values were obtained in the pairwise analysis for the markers that showed a significant result for the association between the functional hallmarks (impulse oscillometry system, spirometry, and FeNO) tests and the COVID-19 severity.

a Severe ≠ mild–moderate (*p* = 0.007).

b Critical ≠ mild–moderate (*p* = 0.002).

c Critical ≠ mild–moderate (*p* = 0.002).

d Critical ≠ severe (*p* = 0.046) and critical ≠ mild–moderate (*p* < 0.001).

e Pairwise showed no statistical significance between groups.

f Critical ≠ mild–moderate (*p* = 0.009).

g Critical ≠ mild–moderate (*p* = 0.002) and severe ≠ mild–moderate (*p* = 0.004).

h Critical ≠ mild–moderate (*p* = 0.002) and severe ≠ mild–moderate (*p* = 0.011).

i Severe ≠ mild–moderate (*p* = 0.009).

j Severe ≠ mild–moderate (*p* = 0.047).

k Severe ≠ mild–moderate (*p* = 0.024).

**Figure 2:**
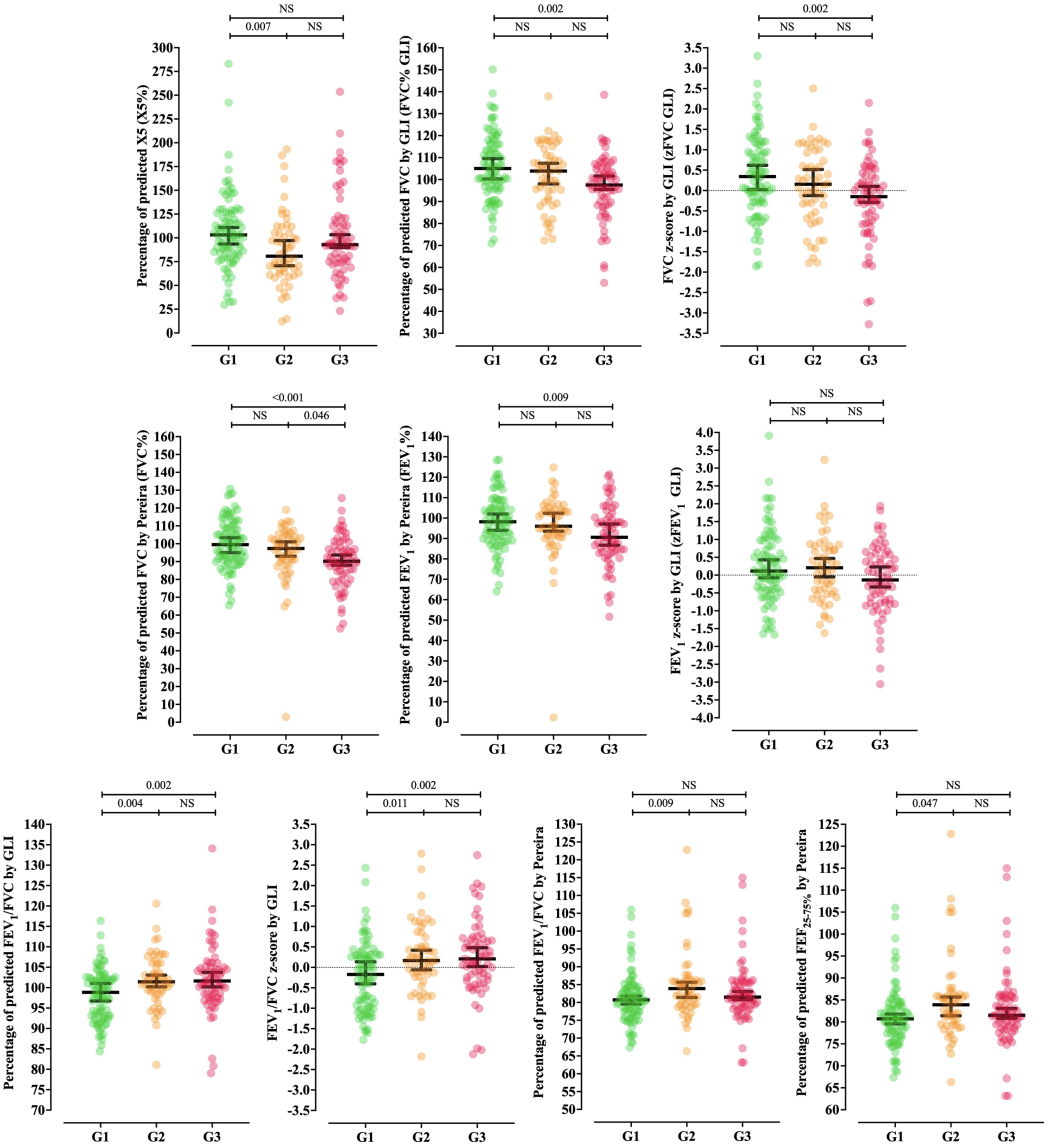
Distribution of lung function variables differing among coronavirus disease (COVID)-19 severity groups. Percentage of predicted reactance at 5 Hz (X5%); percentage of predicted forced vital capacity (FVC) by GLI (FVC% GLI**—**Global Lung Function Initiative); FVC z-score by GLI (zFVC GLI); percentage of predicted FVC by Pereira (FVC% Pereira); forced expiratory volume in 1 s (FEV_1_) z-score by GLI (zFEV_1_ GLI); percentage of predicted FEV_1_/FVC by GLI (FEV_1_/FVC% GLI); FEV_1_/FVC z-score by GLI (zFEV_1_/FVC GLI); percentage of predicted FEV_1_/FVC by Pereira (FEV_1_/FVC% Pereira); percentage of predicted FEF_25–75_ by Pereira (FEF_25–75%_); not significant (NS). Disease severity was determined by a physician, according to the World Health Organization criteria. The G1 phenotype was characterized by mild-to-moderate clinical symptoms, with possible radiological findings or the presence of pneumonia on chest computed tomography. The G2 phenotype was characterized by respiratory distress and/or hypoxia/hypoxemia, whereas the G3 phenotype involved respiratory failure requiring mechanical ventilation, shock, and/or multiorgan dysfunction (5, 16).

**Figure 3:**
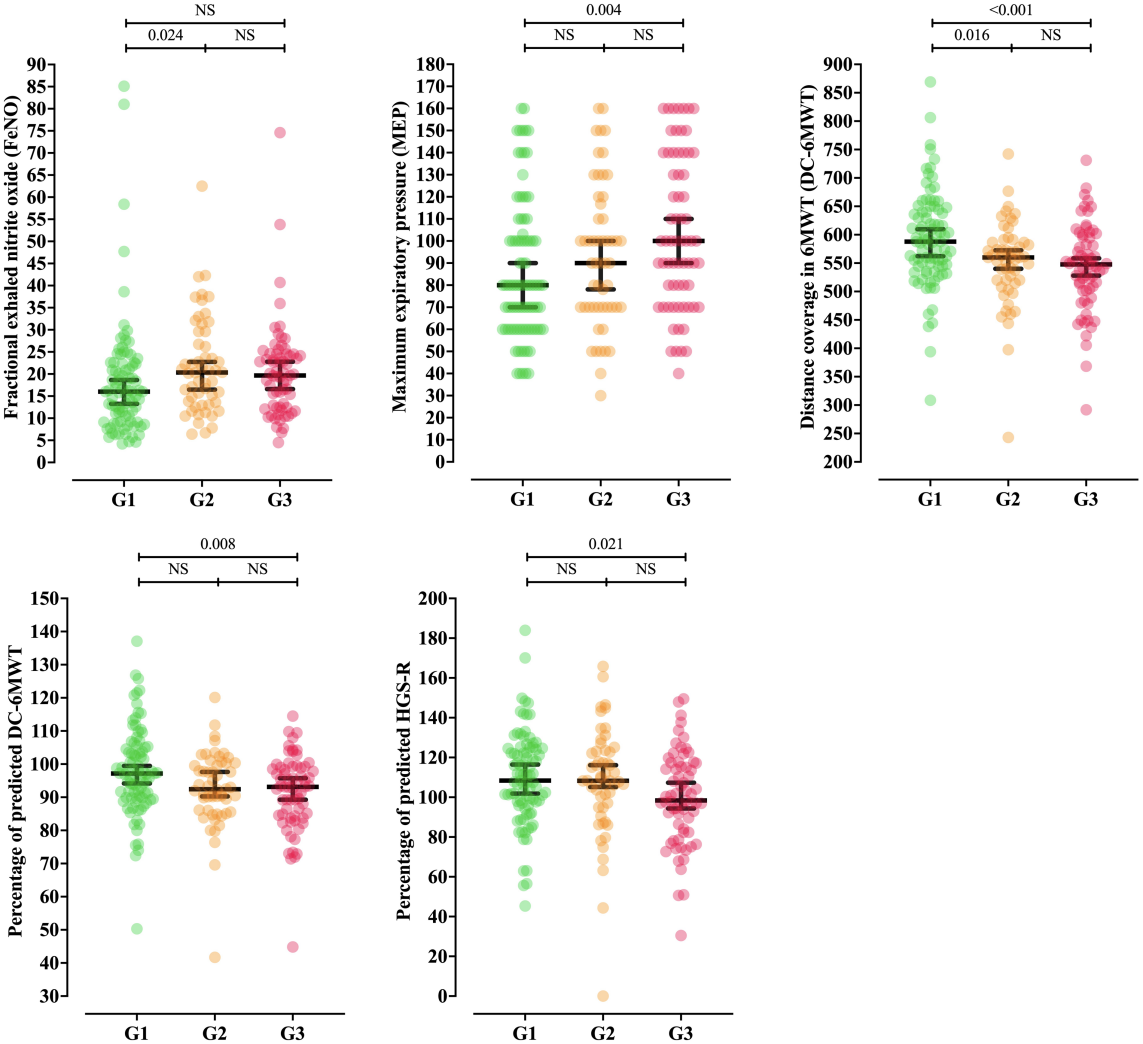
Distribution of functional test variables differing among coronavirus disease (COVID)-19 severity groups. Fractional exhaled nitric oxide (FeNO); maximum expiratory pressure (MEP); distance covered in the 6-min walk test (DC-6MWT); percentage of predicted DC (DC-6MWT%); right-hand grip strength (HGS-R); not significant (NS). Disease severity was determined by a physician, according to the World Health Organization criteria. The G1 phenotype was characterized by mild-to-moderate clinical symptoms, with possible radiological findings or the presence of pneumonia on chest computed tomography. The G2 phenotype was characterized by respiratory distress and/or hypoxia/hypoxemia, whereas the G3 phenotype involved respiratory failure requiring mechanical ventilation, shock, and/or multiorgan dysfunction (5, 16).

*Kruskal–Wallis test; **Analysis of variance (ANOVA). In Figures 2, 3, the significant *p*-values are presented in bold type. Impedance at 5 Hz (Z5); resistance at 5 Hz (R5); percentage of predicted R5 (R5%); resistance at 20 Hz (R20); percentage of predicted R20 (R20%); reactance at 5 Hz (X5); percentage of predicted X5 (X5%); resonance frequency (Fres); percentage of predicted Fres (Fres%); reactance area (AX); percentage of predicted AX (AX%); forced vital capacity (FVC); percentage of predicted FVC by GLI (FVC% GLI—Global Lung Function Initiative); FVC z-score by GLI (zFVC GLI); percentage of predicted FVC by Pereira (FVC% Pereira); forced expiratory volume in 1 s (FEV_1_); percentage of predicted FEV_1_ by GLI (FEV_1_% GLI); FEV_1_ z-score by GLI (zFEV_1_ GLI); percentage of predicted FEV_1_ by Pereira (FEV_1_% Pereira); Tiffeneau index (FEV_1_/FVC); percentage of predicted FEV_1_/FVC by GLI (FEV_1_/FVC% GLI); FEV_1_/FVC z-score by GLI (zFEV_1_/FVC GLI); percentage of predicted FEV_1_/FVC by Pereira (FEV_1_/FVC% Pereira); forced expiratory flow from 25 to 75% of FVC (FEF_25–75_); percentage of predicted FEF_25–75_ by Pereira (FEF_25–75%_). Disease severity was determined by a physician, according to the World Health Organization criteria. The G1 phenotype was characterized by mild-to-moderate clinical symptoms, with possible radiological findings or the presence of pneumonia on chest computed tomography. The G2 phenotype was characterized by respiratory distress and/or hypoxia/hypoxemia, whereas the G3 phenotype involved respiratory failure requiring mechanical ventilation, shock, and/or multiorgan dysfunction (5, 16).

The predicted percentages of FVC of spirometry by the GLI [105.03 (95%CI = 100.65–108.54) vs. 97.53 (95%CI = 95.57–101.61)] (*p* = 0.002) and FVC by the GLI z-score [0.34 (95%CI = 0.04–0.56) vs. −0.15 (95%CI = −0.29 to −0.10)] (*p* = 0.002) were higher in G1 than G3. Additionally, the FVC by Pereira was lower in G3 [90.20 (95%CI = 88.50–93.70)] than G1 [99.50 (95%CI = 95.50–103.30)] (*p* < 0.001) and G2 [97.35 (95%CI = 93.00–101.10)] (*p* = 0.046). Moreover, the percentage of predicted FEV_1_ by Pereira was lower in G3 [90.60 (95%CI = 86.80–97.05)] than G1 [98.20 (95%CI = 94.00–101.10)] (*p* = 0.009).

The Tiffeneau index also showed differences among the severity groups when comparing the percentage of predicted values by GLI—G3 [101.64 (95%CI = 100.30–103.77)] vs. G1 [98.85 (95%CI = 96.93–100.64)] (*p* = 0.002) and G2 [101.42 (95%CI = 100.21–103.05)] vs. G1 (*p* = 0.004), the z-score by GLI — G3 [0.21 (95%CI = 0.04–0.48)] vs. G1 [−0.17 (95%CI = −0.40–0.08)] (*p* = 0.002) and G2 [0.17 (95%CI = −0.05–0.41)] vs. G1 (*p* = 0.011), and the percentage of predicted values by Pereira — G2 [83.90 (95%CI = 81.40–85.66)] vs. G1 [80.70 (95%CI = 79.67–81.63)] (*p* = 0.009). The percentage of predicted FEF_25-75%_ was higher in G2 [105.90 (95%CI = 101.70–116.01)] than G1 [95.40 (95%CI = 87.70–101.70)] (*p* = 0.047). The distribution of all lung function variables that differed among the COVID-19 severity groups is shown in Figure 2.

FeNO differed between groups, with a lower median observed in G1 [16.00 (95%CI = 13.33–18.40)] than G2 [20.35 (95%CI = 16.58–22.48)] (*p* = 0.024) (Table 2; Figure 3). Structural assessment through chest CT showed an association between fibrotic interstitial abnormalities and G2, but not with the other studied features (Table 3).

**Table 3:** Association between chest computed tomography scores and coronavirus disease (COVID)-19 severity.

Markers	Groups	Clinical phenotype during the acute infection phase			Total	*p*-value
		Mild–moderate (G1) *N* = 89	Severe (G2) *N* = 54	Critical (G3) *N* = 67		
Ground glass						
	Yes	69 (77.5%)	36 (66.7%)	47 (70.1%)	152 (72.4%)	0.331 *
	No	20 (22.5%)	18 (33.3%)	20 (29.9%)	58 (27.6%)	
Consolidation						
	Yes	78 (87.6%)	46 (85.2%)	65 (97.0%)	189 (90.0%)	0.065 *
	No	11 (12.4%)	8 (14.8%)	2 (3.0%)	21 (10.0%)	
Interstitial abnormalities						
	Yes	65 (73.0%)	30 (55.6%)	52 (77.6%)	147 (70.0%)	**0.022** *
	No	24 (27.0%)	24 (44.4%)	15 (22.4%)	63 (30.0%)	
Air trapping						
	Yes	66 (74.2%)	37 (68.5%)	51 (76.1%)	154 (73.3%)	0.637 *
	No	23 (25.8%)	17 (31.5%)	16 (23.9%)	56 (26.7%)	
Total						
	Yes	59 (66.3%)	31 (57.4%)	48 (71.6%)	138 (65.7%)	0.262 *
	No	30 (33.7%)	23 (42.6%)	19 (28.4%)	72 (34.3%)	

*Pearson chi-square. The significant *p*-values are presented in bold type. The data are displayed as the number of individuals (N) and percentage (%). Disease severity was determined by a physician, according to the World Health Organization criteria. The G1 phenotype was characterized by mild-to-moderate clinical symptoms, with possible radiological findings or the presence of pneumonia on chest computed tomography. The G2 phenotype was characterized by respiratory distress and/or hypoxia/hypoxemia, whereas the G3 phenotype involved respiratory failure requiring mechanical ventilation, shock, and/or multiorgan dysfunction (5, 16).

### 3.3 Functional capacity evaluation

Respiratory muscle strength evaluation showed an association between MEP absolute values and COVID-19 severity—G1 [80.00 (95%CI = 70.00–90.00)] vs. G3 [100.00 (95%CI = 90.00–110.00)] (*p* = 0.004), but no association with MIP was observed (Table 4; Figure 3). The 6MWT also showed a difference in the distance covered, in both absolute—G1 [587.85 (95%CI = 563.00–609.56)] vs. G2 [560.00 (95%CI = 540.75–572.02)] (*p* = 0.016) and G1 vs. G3 [547.80 (528.00–558.50)] (*p* < 0.001)—and percentage of predicted values—G1 [97.16 (95%CI = 94.39–99.00)] vs. G3 [93.12 (95%CI = 89.25–95.73)] (*p* = 0.008)—among severity groups (Figure 3).

**Table 4:** Association between functional tests and coronavirus disease (COVID)-19 severity.

Markers	Clinical phenotype during the acute infection phase			*p*-value
	Mild–moderate (G1) (*N* = 89)	Severe (G2) (*N* = 54)	Critical (*N* = 67)	
MIP	−100.00 (−110.00 to −90.00)	−125.00 (−140.00 to −100.00)	−110.00 (−120.00 to −100.00)	0.278 *
MIP%	85.52 (80.18–91.05)	90.86 (81.19–99.83)	83.66 (77.71–89.85)	0.345 **
MEP	80.00 (70.00–90.00)	90.00 (80.00–100.00)	100.00 (90.00–110.00)	**0.006** ^**a**^,*
MEP%	79.71 (72.52–88.23)	85.66 (76.00–92.00)	92.76 (81.04–102.48)	0.199 *
DC-6MWT	587.85 (563.00–609.56)	560.00 (540.75–572.02)	547.80 (528.00–558.50)	**< 0.001** ^**b**^,*
DC-6MWT%	97.16 (94.39–99.00)	92.41 (90.27–97.64)	93.12 (89.25–95.73)	**0.006** ^**c**^^, *^
%maxHR	64.13 (61.50–66.83)	65.00 (62.17–67.89)	63.78 (60.81–66.64)	0.850 **
∆HRf-i	37.45 (33.47–41.56)	37.92 (34.38–41.52)	34.04 (29.58–38.63)	0.379 **
∆HRrec	−18.00 (−22.00–-15.96)	−16.64 (−22.00 to −14.00)	−17.00 (−19.58–-14.00)	0.730 *
HGS-R	32.60 (29.70–34.30)	34.95 (30.10–42.00)	34.80 (30.70–38.55)	0.575 *
HGS-R%	108.39 (102.66–116.06)	108.31 (105.73–116.00)	98.38 (94.35–107.29)	**0.021** ^**d**^,*
HGS-L	29.60 (28.80–33.00)	33.15 (27.91–39.60)	33.40 (30.60–37.00)	0.596 *
HGS-L%	111.74 (105.86–115.94)	107.94 (103.11–115.71)	99.76 (93.66–107.56)	0.056 *

The following significant *p*-values were obtained in the pairwise analysis for the markers that showed a significant result for the association between the functional hallmarks (maximum inspiratory pressure, maximum expiratory pressure, 6-min walk test and hand-grip strength) tests and the COVID-19 severity.

a Critical ≠ mild–moderate (*p* = 0.004).

b Critical ≠ mild–moderate (*p* < 0.001) and ≠severe (*p* = 0.016).

c Critical ≠ mild–moderate (*p* = 0.008).

d Critical ≠ mild–moderate (*p* = 0.031).

*Kruskal-Wallis; **Analysis of variance (ANOVA). The significant *p*-values are presented in bold type and in Figure 3. Maximum inspiratory pressure (MIP); percentage of predicted MIP (MIP%); maximum expiratory pressure (MEP); percentage of predicted MEP (MEP%); distance covered in the 6-min walk test (DC-6MWT); percentage of predicted DC (DC-6MWT%); percentage of maximum heart rate (HR) (%maxHR); variation between final HR minus initial HR (∆HRf-i); recovery heart rate (∆HRrec); right-hand grip strength (HGS-R); percentage of predicted HGS-R (HGS-R%); left-hand grip strength (HGS-L); percentage of predicted HGS-L (HGS-L%). Disease severity was determined by a physician, according to the World Health Organization criteria. The G1 phenotype was characterized by mild-to-moderate clinical symptoms, with possible radiological findings or the presence of pneumonia on chest computed tomography. The G2 phenotype was characterized by respiratory distress and/or hypoxia/hypoxemia, whereas the G3 phenotype involved respiratory failure requiring mechanical ventilation, shock, and/or multiorgan dysfunction (5, 16).

The percentage of predicted maximum heart rate, the variation between final and initial heart rate, and the variation in recovery heart rate were not statistically different. Peripheral strength assessment showed a lower median for right-hand grip strength in G3 [98.38 (95%CI = 94.35–107.29)] than G1 [108.39 (95%CI = 102.66–116.06)] (*p*-value = 0.031), with no association observed in the left-hand assessment (Table 4; Figure 3).

A comprehensive summary of the key findings of the study is presented in Graphical Abstract.

## 4 Discussion

The study assessed pulmonary structural and functional characteristics, as well as exercise capacity, at least 3 months after acute COVID-19, according to WHO severity classifications. All the evaluation tools used revealed differences among the severity groups, indicating the persistent effects of SARS-CoV-2 infection. In our study, the key findings include lower predicted FVC and FEV_1_ in G3, along with an increased Tiffeneau index as severity worsened. G2 had higher predicted FEF_25–75%_ and FeNO than G1. Chest CT showed interstitial abnormalities associated with disease severity. MEP increased with severity, whereas the 6MWT distance and right-hand grip strength were lower in G3.

Long COVID-19 syndrome may have varying definitions according to public health institutions of each country. The WHO defines it as the persistence of symptoms and conditions related to COVID-19 for at least 2 months, within 3 months of the onset of COVID-19, and that cannot be explained by an alternative diagnosis (12, 44). Due to the heterogeneity of its symptoms, the diagnosis of long COVID-19, which is strictly clinical, may be uncertain or delayed. Therefore, the detection of sustained pulmonary and functional impairment during the long COVID-19 period, even in mild-to-moderate disease presentations, whether or not combined with clinical symptoms, allows for prompt management and treatment (44). This assessment could help better elucidate some of the mechanisms involved in long COVID-19.

The immune response inherent to COVID-19 may determine the development and severity of the disease. Infected cells release cytokines and chemokines, attracting immune cells to the site of SARS-CoV-2 infection, which causes an overwhelming inflammatory response, damage to the alveoli, edema, and impaired gas exchange, ultimately resulting in tissue damage and pulmonary fibrosis. This event leads to reduced diffusion capacity, which impairs lung function, exercise capacity, and overall functional ability (6). Additionally, studies have found evidence of virus persistence, with viral RNA or proteins detected in various tissues after infection. Sustained inflammation and immune dysfunction could prevent the clearance of residual virus, creating a vicious cycle. The damage caused in some tissues may be irreversible, leading to a chronic inflammatory state, which would explain the persistent symptoms and sustained functional impairment (44). Studies have associated long COVID-19 with interleukin-1β, interleukin-6, and TNF (tumor necrosis factor)-alpha, along with chronic activation of a subset of CD8+ T cells—often called cytotoxic T lymphocytes (44). IOS and spirometry parameters have shown differences among groups, with a later association with diffusion capacity and exercise tolerance months after SARS-CoV-2 infection, which could reflect disease severity (45, 46). These findings are consistent with other studies that detected differences between severe and non-severe groups (46, 47).

Pulmonary fibrosis is an important sequela in patients after severe respiratory disease, and our analysis showed an association between interstitial fibrotic abnormalities and disease severity. Residual abnormalities have been detected in almost half of the evaluated individuals up to a year post-acute SARS-CoV-2 infection, which could be related to persistent symptoms (1, 2, 26). Nevertheless, the literature highlights the limitation in identifying whether the lung damage is entirely due to the viral effect or if it is at least partially secondary to barotrauma and volutrauma during mechanical ventilation in patients with severe and critical phenotypes (27).

Although FeNO is considered an important marker of epithelial damage in the airways during viral infections, and our study found differences between severe and critical groups, the literature highlights that it cannot be used as a biomarker to monitor post-acute infection recovery as eosinophilic airway inflammation may not cause detectable small airway disorders (47, 48). Respiratory muscle strength assessment, on the other hand, can be easily applied as a rehabilitation assessment measure. For example, a post-COVID-19 study showed improvement in MEP values after rehabilitation, highlighting the importance of evaluation and follow-up (49).

The 6MWT has been shown to be a useful rehabilitation monitoring tool, with an increase in the distance covered indicating improved functional capacity and prognosis (50). This study observed shorter distances covered as disease severity increased, suggesting sustained functional capacity impairment after acute SARS-CoV-2 infection, which serves as a predictive parameter for morbidity and mortality (47).

A study conducted exclusively with female participants diagnosed with post-COVID-19 syndrome due to persistent symptoms observed lower hand grip strength in the second set of measurements than individuals with non-post-COVID-19 syndrome and myalgic encephalomyelitis/chronic fatigue syndrome. This finding reflects fatigability and impaired muscle recovery after 1 h. Our study found lower hand grip strength values in critical patients, highlighting the persistent effects of acute disease on later exercise capacity. Therefore, hand grip strength evaluation has proven to be an objective marker of physical function and an important tool for diagnostic and prognostic assessment of multiple systems as well as for rehabilitation monitoring (49, 51).

The COVID-19 pandemic had a significant impact on the global economy, exacerbating poverty and inequality, and overloading Brazil’s public health system (4). The long-term sequelae of SARS-CoV-2 infection—persistent symptoms and post-COVID-19 syndrome—continue to affect socioeconomic factors and quality of life, further straining public health resources (4, 52). In this context, the importance of an individualized, comprehensive, long-term evaluation of COVID-19 survivors becomes clear as it is essential for prompt rehabilitation aimed at preventing persistent complications and improving quality of life.

In the context of our study, a type II error may have impacted the findings in several ways. Here are some considerations:

1. *Underestimation of differences between groups:* If the study did not have sufficient statistical power (which may have been caused by a small sample size or excessive variability in the data), it might not have detected significant differences between the functional variables and the severity of COVID-19, even though such differences truly exist.
2. *Sample size and variability:* If the G1, G2, and G3 groups were not large enough to adequately represent the population, a type II error could have occurred, especially in group comparison tests. Variability in functional markers, such as FVC or the Tiffeneau index, might have led to the failure to detect a true difference between the groups if the sample was small or not well distributed.
3. *Limitations in functional tests:* The use of different functional tests could introduce variability, especially if measurements were not consistent or precise across all participants. If the ability to detect a true difference was impaired by issues in the tests or data interpretation, a type II error could be a concern, suggesting that the tests were not sensitive enough to detect functional differences between the severity groups.
4. *Impact of participants’ clinical conditions:* If the distribution of severity across the groups was not sufficiently balanced, or if some patients had uncontrolled comorbidities that affected the results, there might have been a higher chance of a type II error, leading to the failure to detect differences between the groups.

In summary, a type II error could have occurred in our study, especially if the differences between the COVID-19 severity groups were not detected due to sample size, variability in tests, or measurement inaccuracies. This could suggest that the study did not have sufficient statistical power to detect all true differences, resulting in false-negative results for some markers.

### 4.1 Strengths and limitations of the study

Our study highlighted alterations in pulmonary structure and functional capacity following acute COVID-19, stratifying severity groups according to the WHO classification. This approach differs from other studies that typically divide groups into severe and non-severe phenotypes only. Screening functional assessments across different severity groups helps identify small abnormalities that can be promptly addressed with appropriate and individualized interventions. Additionally, excluding patients with preexisting chronic lung diseases enhances the reliability of the results, in line with the criteria for long COVID-19 syndrome, which requires the absence of an alternative diagnosis for the clinical conditions presented. Our study evaluated the phenotypic profile of patients using a wide range of tools, and concurrently, we obtained the results from the assessment of several markers. The collected dataset provides insight into the complex clinical landscape associated with the progression of patients affected by COVID-19, particularly among those who exhibited clinical signs and symptoms, which were key determining factors in the progression to long COVID-19 syndrome. Finally, Brazil has been facing numerous issues associated with pandemic and epidemic situations (39, 53–56). Therefore, in the future, other studies could be conducted to assess the coexistence of COVID-19, including the presence of long COVID syndrome, alongside other common pandemic and epidemic scenarios in Brazil, such as dengue and influenza infections.

The main limitation of the study was its cross-sectional design, which potentially restricted the amount of information collected. A broader longitudinal study could provide more accurate data on the reversibility of structural abnormalities. The absence of baseline lung function data and previous CT images also limits the analysis of preexisting lung conditions in the participants enrolled in our study. It was not possible to evaluate the genotypic variants of SARS-CoV-2 among the patients included in the study, and despite the high number of participants, we believe that increasing the sample size would be related to an increase in the statistical power of the sample to identify other markers that could potentially differ in relation to the COVID-19 severity groups determined by the use of the WHO clinical severity criteria. In the future, studies similar to those of the NIHR (National Institute for Health and Care Research) initiative should be conducted with the aim of understanding pandemic processes, including those associated with sequelae resulting from the SARS-CoV-2 infection, on a global scale, involving multiple countries in order to improve, especially the sample size and the generalization of the obtained results (57–62).

## 5 Conclusion

Our study highlighted lung functional alterations, impaired functional markers, and a high prevalence of lung abnormalities in post-COVID-19 CT scans, particularly as severity increases. These findings emphasize the complexity of post-COVID-19 functional assessment and the importance of continuous and detailed surveillance by healthcare professionals regarding long-term pulmonary sequelae in patients who have suffered severe forms of the disease. Future studies, with longer follow-up periods, are crucial to elucidate the mechanisms underlying post-COVID-19 sequelae and to develop effective treatment and rehabilitation strategies.

## UNICOVID Study Group (Unicamp Coronavirus Disease Study Group)

Adyléia Aparecida Dalbo Contrera Toro (ORCID ID: orcid.org/0000-0001-5777-1146), Aline Cristina Gonçalves (ORCID ID: orcid.org/0000-0002-2008-1747), Andrea de Melo Alexandre Fraga (ORCID ID: orcid.org/0000-0002-9979-0289), André Moreno Morcillo (ORCID ID: orcid.org/0000-0002-2088-972X), Andressa Oliveira Peixoto (ORCID ID: orcid.org/0000-0002-8407-4087), Andrei Carvalho Sposito (ORCID ID: orcid.org/0000-0001-7127-2052), Bianca Aparecida Siqueira (ORCID ID: orcid.org/0000-0002-8840-9358), Emília Silva Gonçalves (ORCID ID: orcid.org/0000-0001-5985-5319), Emília Raposo Nascimento (ORCID ID: orcid.org/0000-0003-4431-205X), Gil Guerra Junior (ORCID ID: orcid.org/0000-0002-2991-7678), Jenniffer Tayna Orzechowski Furtado (ORCID ID: orcid.org/0009-0000-9402-9645), Lucas Rodrigues de Moraes (ORCID ID: orcid.org/0009-0006-4973-3901), Maria de Fatima Corrêa Pimenta Servidoni (ORCID ID: orcid.org/0000-0002-5237-7227), Mariana Zorron (ORCID ID: orcid.org/0000-0001-7705-9105), Marcos Tadeu Nolasco da Silva (ORCID ID: orcid.org/0000-0001-8342-1959), Maíra Seabra Assumpção (ORCID ID: orcid.org/0000-0002-6884-5662), Milena Baptistela Grotta (ORCID ID: orcid.org/0000-0002-1483-049X), Renan Marrichi Mauch (ORCID ID: orcid.org/0000-0002-9457-0156), Simone Appenzeller (ORCID ID: orcid.org/0000-0001-5075-4474), Thiago Luís Infanger Serrano (ORCID ID: orcid.org/0000-0002-6853-7897).

## Data Availability

The raw data supporting the conclusions of this article will be made available by the authors, without undue reservation.

## Footnotes

^1^ https://www.xlstat.com
