# Correction: Pulmonary and functional hallmarks after SARS-CoV-2 infection across three WHO severity level-groups: an observational study

**DOI:** 10.3389/fmed.2026.1793266

**Published:** 2026-03-11

**Authors:** Patrícia Blau Margosian Conti, Maria Ângela Gonçalves Oliveira Ribeiro, Carla Cristina Souza Gomez, Aline Priscila Souza, Daniela Souza Paiva Borgli, Eulália Sakano, Mauro Alexandre Pascoa, Silvana Dalge Severino, Tayná Castilho, Fernando Augusto Lima Marson, José Dirceu Ribeiro, UNICOVID Study Group

**Affiliations:** 1 Department of Pediatrics, School of Medical Sciences, University of Campinas, Campinas, Brazil; 2 Department of Ophthalmology-Otorhinolaryngology, School of Medical Sciences, University of Campinas, Campinas, Brazil; 3 LunGuardian Research Group, Epidemiology of Respiratory and Infectious Diseases, Postgraduate Program in Health Sciences, São Francisco University, Bragança Paulista, Brazil; 4 Laboratory of Molecular Biology and Genetics, Postgraduate Program in Health Sciences, São Francisco University, Bragança Paulista, Brazil; 5 Laboratory of Clinical Microbiology and Genetics, Postgraduate Program in Health Sciences, São Francisco University, Bragança Paulista, Brazil

**Keywords:** 6-min walk test, chest computed tomography, fractional exhaled nitric oxide, hand grip strength, impulse oscillometry, lung function, maximum expiratory pressure, maximum inspiratory pressure

In the published article, there was a mistake in Table 1. For both male and female participants, the table currently reports the value 26 (50%). The correct value in both cases should be 27 (50%). The corrected Table 1 appears below.

**Table 1:** Distribution of demographic and clinical characteristics of the study participants according to coronavirus disease (COVID)-19 severity.

**Markers**	**Groups**	**Clinical phenotype during the acute infection phase**			**Total**	***p*-value**
		**Mild-moderate (G1)** ***N*** = **89**	**Severe (G2)** ***N*** = **54**	**Critical (G3)** ***N*** = **67**		
**Sex** ^*^						
	Male	29 (32.6%)	27 (50.0%)	44 (65.7%)	100 (47.6%)	**< 0.001**
	Female	60 (67.4%)	27 (50.0%)	23 (34.2%)	110 (52.4%)	
**Age (years)** ^**^						
		44.11 ± 12.12	50.35 ± 12.03	51.58 ± 10.20	48.09 ± 11.97	**< 0.001**
		43.0 [18.0]	52.0 [14.75]	52.0 [16.0]	49.0 [17.0]	
**Hospitalization—general ward stay** ^***, *a*^						
	No	83 (93.3%)	11 (20.4%)	60 (89.6%)	154 (73.3%)	**< 0.001**
	Yes	6 (6.7%)	43 (79.6%)	7 (10.4%)	56 (26.7%)	
**Hospitalization—intensive care unit stay** ^***, *b*^						
	No	88 (98.9%)	48 (88.9%)	8 (11.9%)	144 (68.6%)	**< 0.001**
	Yes	1 (1.1%)	6 (11.1%)	59 (88.1%)	66 (31.4%)	
**Emergency room/Primary health care unit stay** ^***, *c*^						
	No	85 (95.9%)	52 (96.3%)	66 (98.5%)	203 (96.7%)	0.624
	Yes	4 (4.5%)	2 (3.7%)	1 (1.5%)	7 (3.3%)	

^*^Pearson Chi-square test; ^**^Kruskal–Wallis test; ^***^Fisher's Exact test. The significant p-values are presented in bold type.

The numerical data are displayed as mean ± standard deviation and median [interquartile range]. The categorical data are displayed as number of participants (*N*) and percentage (%).

^a^The period during which patients are admitted to a regular hospital ward for monitoring and treatment of their clinical condition.

^b^The period during which patients are admitted to the intensive care unit for more intensive monitoring and treatment due to the severity of their clinical condition.

^c^The period during which patients are under care in the emergency room or primary health care unit for acute management or initial treatment before being discharged or transferred for further care.

Disease severity was determined by a physician, according to World Health Organization criteria. The G1 phenotype was characterized by mild to moderate clinical symptoms, with possible radiological findings or the presence of pneumonia on chest computed tomography. The G2 phenotype was characterized by respiratory distress and/or hypoxia/hypoxemia, while the G3 phenotype involved respiratory failure requiring mechanical ventilation, shock, and/or multiorgan dysfunction (5, 16).

The original version of this article has been updated.

