# Supplementary material for: Pulmonary and functional hallmarks after SARS-CoV-2 infection across three WHO severity level-groups: an observational study
Source: Front Med (Lausanne). 2025 Apr 7;12:1561387. doi: 10.3389/fmed.2025.1561387 (PMC12010923; doi:10.3389/fmed.2025.1561387)
Supplement: Supplementary file 1 [file Table_1.docx]

**Supplementary Material**

**Title:** Pulmonary and functional hallmarks after SARS-CoV-2 infection across three WHO severity level-groups: an observational study

**Running title:** Post-COVID-19 pulmonary and functional capacity

*Methods* **—** *statistical analysis.*

| **Supplementary table 1.** Results of the analysis of the normality distribution of the quantitative markers. | | | |
| --- | --- | --- | --- |
| **Markers** |  | **Kolmogorov-Smirnov test**  **(p-value)** | **Shapiro-Wilk test**  **(p-value)** |
| Z5 |  | **0.001** | **< 0.001** |
| R5 |  | **0.001** | **< 0.001** |
| R5% |  | **< 0.001** | **< 0.001** |
| R20 |  | **0.003** | **< 0.001** |
| R20% |  | 0.200 | **0.004** |
| X5 |  | **< 0.001** | **< 0.001** |
| X5% |  | **< 0.001** | **< 0.001** |
| Fres |  | **< 0.001** | **< 0.001** |
| Fres% |  | **< 0.001** | **< 0.001** |
| AX |  | **< 0.001** | **< 0.001** |
| AX% |  | **< 0.001** | **< 0.001** |
| FVC |  | **0.049** | **0.001** |
| FVC% GLI |  | **0.015** | 0.221 |
| zFVC GLI |  | **0.039** | 0.239 |
| FVC% Pereira |  | **0.001** | **< 0.001** |
| FEV_1_ |  | 0.200 | **0.005** |
| FEV_1_% GLI |  | 0.200 | 0.202 |
| zFEV_1_ GLI |  | 0.200 | 0.125 |
| FEV_1_% Pereira |  | **0.029** | **< 0.001** |
| FEV_1_/FVC |  | **0.033** | **< 0.001** |
| FEV_1_/FVC% GLI |  | **0.044** | **< 0.001** |
| zFEV_1_/FVC GLI |  | **0.001** | **< 0.001** |
| FEV_1_/FVC% Pereira |  | **< 0.001** | **< 0.001** |
| FEF_25-75_ |  | **0.004** | **0.004** |
| FEF_25-75%_ |  | **0.033** | **0.001** |
| FeNO |  | **< 0.001** | **< 0.001** |
| MIP |  | **< 0.001** | **< 0.001** |
| MIP% |  | 0.200 | 0.203 |
| MEP |  | **< 0.001** | **< 0.001** |
| MEP% |  | **0.002** | **< 0.001** |
| DC-6MWT |  | **0.007** | **< 0.001** |
| DC-6MWT% |  | **0.010** | **< 0.001** |
| %maxHR |  | 0.200 | 0.713 |
| ∆HRf-i |  | 0.200 | 0.078 |
| ∆HRrec |  | **< 0.001** | **< 0.001** |
| HGS-R |  | **0.021** | 0.192 |
| HGS-R% |  | 0.074 | **0.007** |
| HGS-L |  | **0.009** | **0.016** |
| HGS-L% |  | 0.061 | **< 0.001** |

The significant p-values are presented in bold type.

Impedance at 5 Hz (Z5); Resistance at 5 Hz (R5); Percentage of predicted R5 (R5%); Resistance at 20 Hz (R20); Percentage of predicted R20 (R20%); Reactance at 5 Hz (X5); Percentage of predicted X5 (X5%); Resonance frequency (Fres); Percentage of predicted Fres (Fres%); Reactance area (AX); Percentage of predicted AX (AX%); Forced vital capacity (FVC); Percentage of predicted FVC by GLI (FVC% GLI — Global Lung Initiative); FVC z-score by GLI (zFVC GLI); Percentage of predicted FVC by Pereira (FVC% Pereira); Forced expiratory volume in the first second (FEV_1_); Percentage of predicted FEV_1_ by GLI (FEV_1_% GLI); FEV_1_ z-score by GLI (zFEV_1_ GLI); Percentage of predicted FEV_1_ by Pereira (FEV_1_% Pereira); Tiffeneau Index (FEV_1_/FVC); Percentage of predicted FEV_1_/FVC by GLI (FEV_1_/FVC% GLI); FEV_1_/FVC z-score by GLI (zFEV_1_/FVC GLI); Percentage of predicted FEV_1_/FVC by Pereira (FEV_1_/FVC% Pereira); Forced expiratory flow from 25% to 75% of FVC (FEF_25-75_); Percentage of predicted FEF_25-75_ by Pereira (FEF_25-75%_); Fractional exhaled nitric oxide (FeNO); Maximum inspiratory pressure (MIP); Percentage of predicted MIP (MIP%); Maximum expiratory pressure (MEP); Percentage of predicted MEP (MEP%); Distance covered in the 6MWT (DC-6MWT); Percentage of predicted DC (DC-6MWT%); Percentage of maximum heart rate (HR) (%maxHR); Variation between final HR minus initial HR (∆HRf-i); Recovery heart rate (∆HRrec); Right hand grip strength (HGS-R); Percentage of predicted HGS-R (HGS-R%); Left hand grip strength (HGS-L); Percentage of predicted HGS-L (HGS-L%; 6-minute walk test (6MWT).
